# Phase 3 clinical trials evaluating poly(ADP-ribose) polymerase inhibition plus immunotherapy for first-line treatment of advanced ovarian cancer

**DOI:** 10.1093/oncolo/oyaf270

**Published:** 2025-09-17

**Authors:** B J Rimel, Eric Pujade-Lauraine, Kathleen Moore, Jacobus Pfisterer, Sileny Han, David Cibula, Anna Reyners, Andrés Redondo, Christos Papadimitriou, Ram Eitan, Sandro Pignata, Rosalind Glasspool, Mansoor Raza Mirza, Lubomir Bodnar, Linda Duska, Diane Provencher, Rébécca Phaëton, Manjinder Bains, Elif Coskuncay, Anne Claire Hardy-Bessard

**Affiliations:** Division of Gynecologic Oncology, Department of Obstetrics and Gynecology, Cedars-Sinai Medical Center, Los Angeles, CA 90048, United States; Medical Oncology Department, Hôpital Hôtel-Dieu, University of Paris, Paris 75004, France; Department of Obstetrics and Gynecology, Stephenson Cancer Center, University of Oklahoma Health Sciences Center, Oklahoma City, OK 73104, United States; AGO Study Group, Wiesbaden 65185, Germany; Department of Gynecological Oncology, Gynecologic Oncology Center, Kiel 24105, Germany; Department of Gynecologic Oncology, Universitair Ziekenhuis Leuven, Leuven 3000, Belgium; Department of Gynaecology, Obstetrics and Neonatology First Faculty of Medicine, Charles University, Prague 116 36; and General University Hospital in Prague, Prague 128 08, Czech Republic; Department of Medical Oncology, University Medical Center Groningen, University of Groningen, Groningen 9700 RB, The Netherlands; Department of Medical Oncology, Hospital Universitario La Paz-IdiPAZ and GEICO, Madrid 28046, Spain; HeCOG and Oncology Unit, Aretaieion University Hospital, National and Kapodistrian University of Athens, Athens 115 28, Greece; Gynecologic Oncology Division, Rabin Medical Center, Petah Tikva 4941492, Israel; Uro-Gynecologic Oncology Unit, Istituto Nazionale Tumori IRCCS Fondazione G Pascale, Naples 80131, Italy; Beatson West of Scotland Cancer Centre and School of Cancer Sciences, University of Glasgow, Glasgow G12 0YN, United Kingdom; Department of Cancer Treatment, Rigshospitalet—Copenhagen University Hospital, Copenhagen 2100, Denmark; Faculty of Medical and Health Sciences, University of Siedlce, Siedlce 08-110, Poland; Department of Obstetrics and Gynecology, University of Virginia School of Medicine, Charlottesville, VA 22903, United States; Division of Gynecologic Oncology, Université de Montréal, Montreal, Quebec H2X 3E4, Canada; Global Medical Affairs, GSK, Collegeville, PA 19426, United States; Global Medical Affairs, GSK, London W4 2HD, United Kingdom; Global Medical Affairs, GSK, London W4 2HD, United Kingdom; Oncologie Médicale, Centre CARIO —HPCA, and Groupe d’Investigateurs Nationaux pour l’Etude des Cancers Ovariens (GINECO), Plérin Sur Mer, Plérin 22190, France

**Keywords:** advanced ovarian cancer, first-line, maintenance, PARP inhibition, PD-(L)1 inhibition, immunotherapy

## Abstract

**Background:**

Ovarian cancer is the second deadliest gynecologic malignancy globally. The current standard of care first-line therapy for newly diagnosed advanced epithelial ovarian cancer is surgery and platinum-based chemotherapy (±bevacizumab), followed by maintenance therapy with a poly(ADP-ribose) polymerase (PARP) inhibitor, bevacizumab, or a combination of the two. Although anti-programmed cell death (PD) protein 1 and anti–PD ligand 1 antibodies (PD-[L]1 inhibitors) have shown benefit in several solid tumors, their effect in ovarian cancer remains uncertain. Several trials are evaluating PD-(L)1 inhibitors in combination with first-line platinum-based chemotherapy and PARP inhibitor maintenance treatment. Here, we review trial designs to understand key similarities and differences for future assessments of the results.

**Materials and Methods:**

The clinical trials registry “ClinicalTrials.gov” was searched using keywords, including ovarian cancer and niraparib, olaparib, or rucaparib. Search results were then filtered for phase 3 and manually reviewed to identify trials evaluating combinations of PARP inhibitors and PD-(L)1 inhibitors in the first-line setting.

**Results:**

Four trials, ENGOT-OV44/FIRST (NCT03602859), ENGOT-OV46/AGO-OVAR 23/GOG-3025/DUO-O (NCT03737643), ENGOT-OV43/GOG-3036/KEYLYNK-001 (NCT03740165), and ENGOT-OV45/GOG-3020/ATHENA (NCT03522246), were identified. Of these, FIRST, DUO-O, and KEYLYNK-001 are evaluating both first-line use in combination with chemotherapy and maintenance, whereas ATHENA focuses on maintenance after a response to chemotherapy; however, DUO-O and KEYLYNK-001 do not include a PARP inhibitor in the comparator arm, limiting the ability to compare the added benefit of immunotherapy over the current standard of care.

**Conclusions:**

Results of these trials will determine whether PARP inhibitor and PD-(L)1 inhibitor combination with or without bevacizumab can improve patient outcomes.

Implications for Practice Comparison of study designs for phase 3 clinical trials in which combinations of PARP inhibitors and immunotherapies are being evaluated offers important insights into the future treatment paradigms for advanced ovarian cancer. By addressing key stratification factors, such as *BRCA* mutation and homologous recombination deficiency status, and investigating progression-free survival and overall survival outcomes, results of these trials could help determine the role of immunotherapy in the first-line treatment of ovarian cancer. Understanding these trial designs and how they differ is essential for oncologists to better understand how the results of these trials relate to the current standard of care.

## Introduction

Globally, ovarian cancer is the eighth most common cancer in women and accounted for an estimated 3.4% of cases and 4.8% of cancer deaths among women in 2022.^1^ Due to the nonspecific nature of symptoms and the lack of defined screening tests,^2^ ovarian cancer is one of the deadliest gynecologic malignancies.^1^ Although it is a heterogenous group of genetically and etiologically distinct malignancies, epithelial tumors account for more than 90% of ovarian cancers^3–5^ ≈70% of which are high-grade serous carcinomas and up to 15% of which are high-grade endometrioid carcinomas.^6^^,^^7^ Most patients (≈75%) with epithelial ovarian cancer are diagnosed with an advanced stage disease (stage III or IV), for which prognosis is poor and relapse rates are high.^8–10^

The primary treatment for newly diagnosed advanced ovarian cancer involves primary cytoreductive surgery, if the patient is medically able to undergo surgery, followed by adjuvant chemotherapy.^4^ When complete cytoreductive surgery is not feasible or surgery is not feasible due to comorbidities, platinum-based neoadjuvant chemotherapy is administered, and interval cytoreductive surgery (if possible) followed by additional chemotherapy is recommended.^4^ For decades, the standard chemotherapy for primary ovarian cancer has included the combination of carboplatin and paclitaxel.^4^^,^^11^

Despite high response rates with platinum-based chemotherapy, there remains a need for further improvement in outcomes in high-risk ovarian cancer.^12^ In the ICON7 and GOG-0218 trials, the addition of bevacizumab (a monoclonal antibody targeting vascular endothelial growth factor) to carboplatin and paclitaxel, followed by bevacizumab maintenance treatment,^13^^,^^14^ resulted in a statistically significant increase in progression-free survival (PFS).^15^^,^^16^ However, improvement in overall survival (OS) was seen only in the exploratory analyses of the subgroup of patients with stage III disease and unresectable or suboptimally debulked tumors or stage IV disease (ICON7), and those with ascites or stage IV disease (GOG-0218), which are associated with poor prognosis.^4^^,^^17–19^

Germline or somatic mutations in certain homologous recombination genes have been associated with increased sensitivity to platinum-based chemotherapy and poly(ADP–ribose) polymerase (PARP) inhibition.^20^^,^^21^ PARP inhibitors work by trapping PARP on single-strand DNA break sites, thereby preventing their repair and generating double-strand breaks that cannot be repaired accurately in tumors with homologous recombination deficiency (HRD), such as tumors with a mutation in *BRCA1, BRCA2*, or other genes involved in homologous recombination.^22^ Thus, the first approval, in 2018, for a PARP inhibitor (olaparib) in the first-line maintenance setting was for patients with pathogenic *BRCA* mutations who had a complete or partial response to first-line chemotherapy.^23–25^ Olaparib was subsequently approved in 2020, in combination with bevacizumab, for first-line maintenance treatment in patients with HRD-positive advanced ovarian cancer.^23^^,^^24^ In these patients, addition of maintenance olaparib to bevacizumab provided both PFS and OS benefits in patients with HRD-positive tumors, including those without a *BRCA* mutation.^26^^,^^27^ The PARP inhibitor niraparib was also approved in 2020 as first-line maintenance treatment for all patients with advanced ovarian cancer after demonstrating significant PFS benefit, regardless of HRD status.^24^^,^^28^^,^^29^ However, in OS analyses, there was no observed difference between niraparib first-line maintenance and placebo treatment arms, regardless of HRD status.^30^ In 2023, the PARP inhibitor rucaparib was approved by the European Medicines Agency (EMA) as first-line maintenance treatment for all patients with advanced ovarian cancer after demonstrating significant PFS benefit in patients with and without HRD-positive tumors; however, it is not approved by the US Food and Drug Administration for this indication (first-line maintenance indications of PARP inhibitors for ovarian cancer are shown in Table 1).^31–33^ In interim OS analyses for first-line maintenance rucaparib treatment of patients with newly diagnosed advanced ovarian cancer, OS data were immature at the time of data cutoff, with the exception of the placebo arm of the intention-to-treat population; mature OS data have not yet been reported.^33^^,^^34^

**Table 1.** Current first-line maintenance indications of PARP inhibitors for ovarian cancer.^a^

	United States	European Union
**Niraparib**	Maintenance treatment of adult patients with advanced epithelial ovarian, fallopian tube, or primary peritoneal cancer who are in a complete or partial response to first-line platinum-based chemotherapy	Monotherapy for the maintenance treatment of adult patients with advanced epithelial (FIGO stages III and IV) high-grade ovarian, fallopian tube, or primary peritoneal cancer who are in response (complete or partial) after completion of first-line platinum-based chemotherapy
**Olaparib**	Maintenance treatment of adult patients with deleterious or suspected deleterious germline or somatic *BRCA*-­mutated advanced epithelial ovarian, fallopian tube, or primary peritoneal cancer who are in complete or partial response to first-line platinum-based chemotherapy In combination with bevacizumab for the maintenance treatment of adult patients with advanced epithelial ovarian, fallopian tube, or primary peritoneal cancer who are in complete or partial response to first-line platinum-based chemotherapy and whose cancer is associated with HRD-­positive status defined by either a deleterious or suspected deleterious *BRCA* mutation, and/or genomic instability	Monotherapy for the maintenance treatment of adult patients with advanced (FIGO stages III and IV) *BRCA1/2*-mutated (germline and/or somatic) high-grade epithelial ovarian, fallopian tube, or primary peritoneal cancer who are in response (complete or partial) after completion of first-line platinum-based chemotherapy In combination with bevacizumab for the maintenance treatment of adult patients with advanced (FIGO stages III and IV) high-grade epithelial ovarian, fallopian tube, or primary peritoneal cancer who are in response (complete or partial) after completion of first-line platinum-based chemotherapy in combination with bevacizumab and whose cancer is associated with HRD-positive status defined by either a *BRCA1/2* mutation and/or genomic instability
**Rucaparib**	Rucaparib does not have an approval in the first-line setting in the US	Monotherapy for the maintenance treatment of adult patients with advanced (FIGO stages III and IV) high-grade epithelial ovarian, fallopian tube, or primary peritoneal cancer who are in response (complete or partial) after completion of first-line platinum-based chemotherapy

Abbreviations: FIGO, International Federation of Gynecology and Obstetrics; HRD, homologous recombination deficient; PARP, poly(ADP–ribose) polymerase.

a Information in this table was restricted to the first-line setting for ovarian cancer for simplicity; these drugs may be approved in other indications not listed here.

Incorporation of PARP inhibitors as maintenance treatment after first-line chemotherapy introduced a new era in the management of advanced ovarian cancer. Current standards of care in advanced epithelial ovarian cancer include first-line chemotherapy (±bevacizumab) followed by maintenance treatment with the PARP inhibitors olaparib (±bevacizumab), niraparib, or rucaparib (EMA approval only), or bevacizumab alone (for *BRCA* wild-type [*BRCA*wt]/HRD-negative ovarian cancer), in patients who have a response to chemotherapy.^4^ However, despite substantial benefits with first-line treatment and maintenance therapy, a majority of patients will experience relapse,^25^^,^^35^ highlighting the need for additional treatment options to improve outcomes in patients with advanced ovarian cancer.

Chronic PARP inhibition has been shown to cause sustained DNA damage that promotes several cellular mechanisms, including increased genomic instability, immune pathway activation through generation of neoantigens, and increased programmed cell death ligand 1 (PD-L1) expression on cancer cells, that can promote responsiveness to immune checkpoint inhibitors.^36^ Immune checkpoint inhibitors have revolutionized cancer treatment and are currently incorporated in the management of many solid tumors.^37^^,^^38^ Antibodies targeting the checkpoint proteins programmed cell death protein 1 (PD-1) and PD-L1 (collectively referred to as PD-[L]1 inhibitors in this review) have become some of the most widely prescribed anticancer therapies.^38^^,^^39^ In the first-line treatment setting for ovarian cancer, avelumab (anti–PD-L1 antibody) showed no PFS benefits vs. placebo either in combination with chemotherapy or as a maintenance treatment in the randomized, controlled, phase 3 JAVELIN Ovarian 100 trial, which was eventually stopped for crossing the prespecified futility boundaries.^40^ Likewise, in the placebo-controlled, double-blind, randomized, phase 3 IMagyn050/GOG 3015/ENGOTOV39 trial, addition of atezolizumab (anti–PD-L1 antibody) to platinum-based chemotherapy and bevacizumab did not improve PFS and OS in the intention-to-treat or PD-L1–positive populations, though survival benefits were seen in post hoc exploratory analyses of patients with high (≥5%) PD-L1 immune cell expression.^41^^,^^42^

While PD-(L)1 inhibitors did not show survival benefits as a single agent or in combination with chemotherapy in patients with recurrent ovarian cancer in phase 3 clinical trials,^43–47^ this is hypothesized to have resulted from the more highly immunosuppressive tumor microenvironment in the recurrent setting than that in the chemonaïve setting.^48^ The rationale for combining PARP inhibitors with immune checkpoint inhibitors was supported by preclinical data, which suggest that a combination of PD-(L)1 inhibitors with PARP inhibitors may result in improved activity.^36^^,^^49^ The initial clinical trial data for immune checkpoint inhibitors in combination with PARP inhibitors have been mixed. In the recurrent late-relapsing ovarian cancer setting, the combination of atezolizumab plus platinum-based chemotherapy with maintenance niraparib showed no significant improvement in PFS in the phase 3 ANITA trial,^50^ whereas the open-label, phase 2, basket trial, MEDIOLA, had promising results. The MEDIOLA trial showed high objective response rates with durvalumab (anti–PD-L1 antibody) and olaparib in patients with germline *BRCA*-mutated (g*BRCA*m), platinum-sensitive recurrent ovarian cancer and benefit of durvalumab-olaparib-bevacizumab triple therapy in patients with non–g*BRCA*m platinum-sensitive recurrent ovarian cancer.^51^ Results of studies have shown that chemotherapy can potentiate immunogenicity of the primary tumors, whereas recurrent tumors are characterized by immunosuppressed tumor microenvironment.^52^ Therefore, further investigation of PD-(L)1 inhibitors and PARP inhibitors with platinum-based chemotherapy in the first-line setting was warranted for patients with advanced ovarian cancer.

Currently, several phase 3 trials are evaluating standard first-line chemotherapy (carboplatin and paclitaxel ±bevacizumab) plus a PD-(L)1 inhibitor and a PARP inhibitor plus a PD-(L)1 inhibitor (±bevacizumab) as first-line maintenance treatment. There are variations in the study designs that have potential implications for the results. Therefore, to aid in future evaluation of results from these trials, we conducted a review of the trial designs to understand key similarities and differences between these trials.

## Materials and methods

### Clinical trial identification

A search of ClinicalTrials.gov was performed on May 14, 2024, using “ovarian cancer” as the “condition/disease” and “niraparib,” “olaparib,” or “rucaparib” as the “intervention/treatment.” Results were then filtered for “phase 3” trials that were “not yet recruiting,” “recruiting,” “active, not recruiting,” or “completed or terminated.” After application of filters, 8 trials were identified for niraparib, 12 for olaparib, and 2 for rucaparib. These results were reviewed manually to identify trials evaluating PARP inhibitors in combination with a PD-(L)1 inhibitor in the first-line setting. Trials not focused on first-line treatment, or those without immunotherapy as a component, were excluded. Four trials satisfied our inclusion criteria: 1 for niraparib (ENGOT-OV44/FIRST [NCT03602859]^53^), 2 for olaparib (ENGOT-OV46/AGO-OVAR 23/GOG-3025/DUO-O [NCT03737643]^54^ and ENGOT-OV43/GOG-3036/KEYLYNK-001 [NCT03740165]^55^), and 1 for rucaparib (ENGOT-OV45/GOG-3020/ATHENA [NCT03522246]^56^).

All information on the trials in this review is publicly available and/or published. No outcomes data will be presented or discussed.

## Trial design elements

### General trial information

All 4 trials included here are international, double-blinded, randomized, controlled phase 3 trials. At the time of writing, all were ongoing but had completed recruitment (general information for the trials is shown in Table 2; key trial protocol updates after registration on ClinicalTrials.gov are shown in Table S1); DUO-O and ATHENA had reported PFS results.^57^^,^^58^

**Table 2.** General trial information for phase 3 trials evaluating PARP inhibitors plus immunotherapy for the first-line treatment of advanced ovarian cancer.

	First-line and first-line maintenance treatment settings			First-line maintenance treatment setting only
	FIRST (NCT03602859)^53^	DUO-O (NCT03737643)^54^	KEYLYNK-001 (NCT03740165)^55^	ATHENA (NCT03522246)^56^ ^,^ ^57^ ^,^ ^58^
**Trial characteristics**	Phase 3 randomized	Phase 3 randomized	Phase 3 randomized	Phase 3 randomized
**Countries with research sites**	Americas: Canada, United States Europe: Belarus, Belgium, Czechia, Denmark, Finland, France, Germany, Greece, Israel, Italy, Netherlands, Norway, Poland, Romania, Spain, Ukraine, United Kingdom	Americas: Brazil, Canada, Peru, United States Europe: Austria, Belgium, Bulgaria, Denmark, Finland, France, Germany, Hungary, Italy, Poland, Romania, Spain, Turkey Western Pacific: China, Japan, Republic of Korea	Africa: South Africa Americas: Brazil, Canada, Chile, Colombia, United States Europe: Belgium, Czechia, France, Germany, Hungary, Israel, Italy, Poland, Russian Federation, Spain, Turkey, Ukraine Western Pacific: Australia, Japan, Republic of Korea, Taiwan	Americas: Canada, United States Europe: Belgium, Czechia, Denmark, Finland, Germany, Greece, Ireland, Israel, Italy, Poland, Romania, Russian Federation, Spain, Sweden, Turkey, United Kingdom Western Pacific: Australia, Japan, New Zealand, Republic of Korea, Singapore, Taiwan
**Masking**	Triple (patient, care provider, investigator)	Quadruple (patient, care provider, investigator, outcomes assessor)	Quadruple (patient, care provider, investigator, outcomes assessor)	Quadruple (patient, care provider, investigator, outcomes assessor)
**Estimated primary completion date**	October 25, 2024	March 28, 2025	August 26, 2024 (actual)	December 30, 2024
**Treatment line**	First-line	First-line	First-line	First-line maintenance
**Timing of randomization**	After 1 cycle of platinum-based chemotherapy run-in	After 1 cycle of platinum-­based chemotherapy ± bev^60^	After 1 cycle of platinum-­based chemotherapy run-in	Before first-line maintenance therapy
**No. of treatment arms**	1 Comparator arm 1 Experimental arm^a^	1 Comparator arm 3 Experimental arms^b^	1 Comparator arm 2 Experimental arms	1 Comparator arm 3 Experimental arms
**Original estimated enrollment, *n***	960^c^	1056^d^	1086^e^	1012^f^

Abbreviations: bev, bevacizumab; PARP, poly(ADP–ribose) polymerase; t*BRCA*m, tumor *BRCA-*mutated.

a The standard of care + placebo arm in the FIRST trial was cancelled and enrollment discontinued after admission of 193 patients. These patients were removed from the blinding and offered niraparib maintenance.

b One of the study arms comprises the t*BRCA*m cohort, which will receive the same treatment as 1 of the experimental treatment arms (platinum-based chemotherapy in combination with bev and durvalumab followed by maintenance bev, durvalumab, and olaparib. Bev is optional according to local practice).

c Actual enrollment as of July 31, 2023, was 1402 patients.

d Actual enrollment as of October 31, 2024, was 1407 patients.

e Actual enrollment as of November 16, 2022, was 1367 patients.

f Estimated enrollment was updated to 1000 on October 29, 2020, and a total of 863 patients were randomly assigned to treatment arms between August 7, 2018, and October 26, 2020.^57^

According to the records on ClinicalTrials.gov, each trial had an estimated original enrollment of approximately 1000 patients (Table 2).^53–56^ Although all trials address the efficacy of a combination of a PARP inhibitor and a PD-(L)1 inhibitor, the study designs differ, which may affect the outcomes and their interpretation. FIRST, DUO-O, and KEYLYNK-001 evaluate the full first-line treatment regimen, including first-line use in combination with chemotherapy and maintenance therapy, whereas ATHENA evaluates the maintenance setting. In FIRST and KEYLYNK-001, randomization occurred after the first chemotherapy cycle (after the run-in period).^59^ In DUO-O, randomized patients had either completed upfront debulking surgery or planned to receive interval debulking surgery and had completed 1 cycle of chemotherapy ±bevacizumab.^60^ In ATHENA, patients were included after a complete or partial response to first-line platinum-based chemotherapy and surgery. This key difference may result in a more homogeneous and platinum-sensitive (which is prognostically more favorable) patient population in ATHENA, potentially influencing the outcomes^61^; in FIRST, DUO-O, and KEYLYNK-001, patient randomization after 1 cycle of chemotherapy will allow assessment of efficacy for PARP inhibitor and immunotherapy across a broader population. In addition, these differences in study design may make comparisons with the findings from ATHENA particularly difficult because FIRST, DUO-O, and KEYLYNK-001 enroll patients who would receive chemotherapy as part of the protocol, so sensitivity to chemotherapy is unknown.

### Patient populations

**Figure 1.**
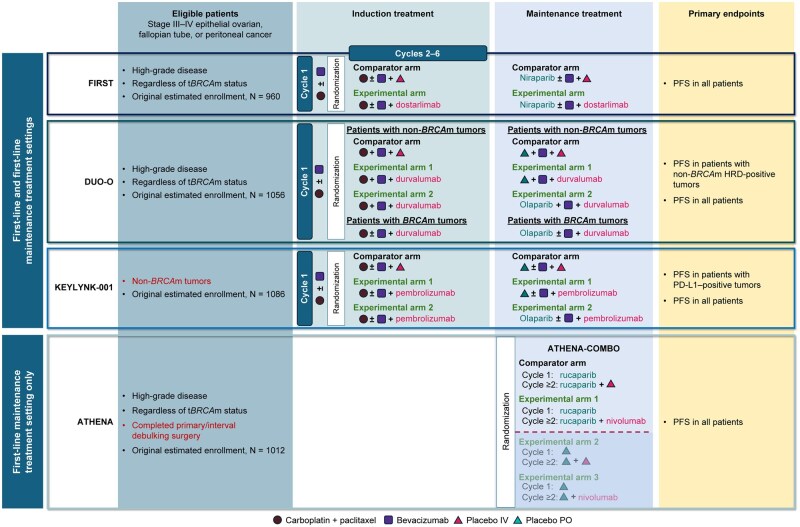
General trial information for phase 3 trials evaluating PARP inhibitors plus immunotherapy for the first-line treatment of advanced ovarian cancer. Abbreviations: *BRCA*m, *BRCA*-mutated; HRD, homologous recombination deficiency; IV, intravenously; PARP, poly(ADP–ribose) polymerase; PD-L1, programmed cell death-ligand 1; PFS, progression-free survival; PO, orally; t*BRCA*m, tumor *BRCA*-mutated.

Patients in all 4 trials have stage III or IV epithelial ovarian, fallopian tube, or peritoneal cancer (collectively referred to as “ovarian cancer”; Table 3 and Figure 1). All trials exclude patients with mucinous tumors, a rare ovarian cancer histologic type that responds poorly to chemotherapy and targeted therapies.^62^ Individuals with central nervous system involvement are also excluded in all trials. Presence of high-grade epithelial carcinoma is a requirement in FIRST, DUO-O, and ATHENA but not in KEYLYNK-001, which allows patients with any-grade endometrioid and low-grade serous ovarian cancer (which combined account for as much as 17% of epithelial ovarian cancer cases).^63^

**Table 3.** Key eligibility criteria in phase 3 trials evaluating PARP inhibitors plus immunotherapy for the first-line treatment of advanced ovarian cancer.

	First-line and first-line maintenance treatment settings			First-line maintenance treatment setting only
	FIRST (NCT03602859)	DUO-O (NCT03737643)	KEYLYNK-001 (NCT03740165)	ATHENA (NCT03522246)
**Disease characteristics**				
** Stages III and IV epithelial ovarian, fallopian tube, or peritoneal cancer**	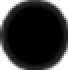	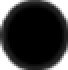	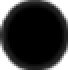	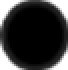
** Staging system**	FIGO/TNM	FIGO	FIGO	FIGO
** High-grade disease**	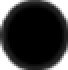	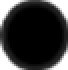	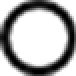 ^a^	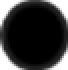
** Non-*BRCA*m tumors**	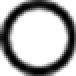	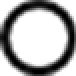	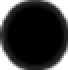	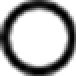
** Mucinous tumors**	**X**	**X**	**X**	**X**
**Cytoreductive surgery requirement**	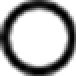	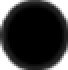	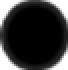	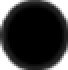
** Completion of cytoreductive surgery pre-enrollment**	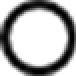	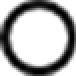	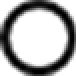	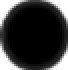
** Completed/eligible for primary debulking surgery**	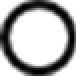	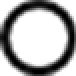	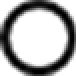	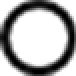
** Eligible for interval debulking surgery**	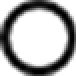	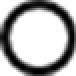	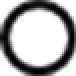	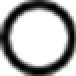
**Responded to first-line platinum-based chemotherapy and surgery**	N/A^b^	N/A^b^	N/A^b^	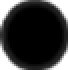

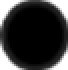
 indicates required; 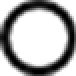 indicates allowed but not required; X indicates excluded; N/A indicates not applicable.

Abbreviations: *BRCA*m, *BRCA*-mutated; FIGO, International Federation of Gynecology and Obstetrics; PARP, poly(ADP–ribose) polymerase; TNM, tumor, node, and metastasis.

a Eligible patients had stage III or IV epithelial ovarian cancer (high-grade predominantly serous, any grade endometrioid, carcinosarcoma, mixed Müllerian with high-grade serous component, clear cell, or low-grade serous ovarian cancer), primary peritoneal cancer, or fallopian tube cancer.

b First-line platinum-based chemotherapy and surgery are evaluated during the trial.

Patients in all 4 trials are required to provide a tumor sample for biomarker analysis, including tumor *BRCA1/2* status and/or PD-L1 levels. FIRST, DUO-O, and ATHENA enroll all patients regardless of tumor *BRCA* status; however, DUO-O randomizes patients with *BRCA* wild-type on tumor testing (t*BRCA*wt) cancer separately in the blinded part of the trial, whereas patients with *BRCA-*mutated on tumor testing (t*BRCA*m) cancer are enrolled in an open-label cohort to ensure the patients who are most likely to benefit from PARP inhibitor treatment receive it.^54^^,^^60^ KEYLYNK-001 only enrolls patients with non-*BRCA*m disease. These differences can affect the interpretation of efficacy across different subgroups and may influence future biomarker-driven treatment approaches.

Of the 4 trials, only ATHENA requires patients to have completed either primary or interval debulking surgery before enrollment. Additionally, FIRST enrolls patients who are at a higher risk of disease progression because patients with stage III disease who had no evidence of macroscopic disease (R0) after primary debulking surgery are excluded unless they have aggregate ≥5 cm extrapelvic disease during primary debulking surgery, per investigator assessment. Patients with stage III disease are also enrolled if they have inoperable disease, macroscopic residual tumor after primary debulking surgery, or are going to receive neoadjuvant chemotherapy. Thus, although the exact makeup of the patient population and its effect on outcomes for each trial will not be known until results are published, upfront differences in patient eligibility criteria can give insights on ways the populations may differ.

### Stratification factors

Stratification factors play a crucial role in the interpretation of trial results, particularly in the context of biomarker-driven therapies, and varied across the 4 trials (Table 4). *BRCA* mutation status is used as a stratification factor in FIRST and ATHENA. FIRST stratifies randomization based on homologous recombination repair (HRR) mutation status (ie, *BRCA*m, *BRCA*wt HRR positive, and *BRCA*wt HRR negative or not determined), whereas ATHENA stratifies based on HRD status (*BRCA*m, *BRCA*wt/loss of heterozygosity [LOH] high [≥16%], *BRCA*wt/LOH low [<16%], or *BRCA*wt/LOH indeterminate). These factors are likely to influence PFS and OS outcomes, with patients with HRD-positive tumors expected to benefit more from PARP inhibitor therapy. PD-L1 expression is a stratification factor only in KEYLYNK-001 (tumor PD-L1 combined positive score of <10 or ≥10).

**Table 4.** Randomization stratification factors in phase 3 trials evaluating PARP inhibitor plus immunotherapy for the first-line treatment of advanced ovarian cancer.

	First-line and first-line maintenance treatment settings			First-line maintenance treatment setting only
	FIRST (NCT03602859)	**DUO-O** ^58^ ** (NCT03737643)**	**KEYLYNK-001** ^59^ ** (NCT03740165)**	**ATHENA** ^65^ ** (NCT03522246)**
**Timing of randomization**	After 1 cycle of platinum-­based chemotherapy run-in	After 1 cycle of platinum-based chemotherapy ± bev^60^	After 1 cycle of platinum-­based chemotherapy run-in	Before first-line maintenance therapy
**Randomization stratification factors**				
** Concurrent bev use**	✓		✓	
** HRR mutation status^a^**	✓			
***BRCA* and/or HRD status^b^**				✓
** Disease burden^c^**	✓			
** Surgical status^d^**		✓	✓	
** Region^e^**		✓		
** PD-L1 combined positive score^f^**			✓	
**Response to first-line platinum-­based chemotherapy^g^**				✓
** Type of debulking surgery^h^**				✓

Abbreviations: bev, bevacizumab; *BRCA*m, *BRCA-*mutated; *BRCA*wt, *BRCA* wild-type; HRD, homologous recombination deficiency; HRR, homologous recombination repair; LOH, loss of heterozygosity; PARP, poly(ADP–ribose) polymerase; PD-L1, programmed cell death-ligand 1.

a Categories included tumor status of *BRCA*m, *BRCA*wt HRR positive, *BRCA*wt HRR negative/not determined.

b Categories included tumor status *BRCA*m, *BRCA*wt/high LOH, *BRCA*wt/low LOH, or *BRCA*wt/LOH indeterminate.

c Defined as the presence vs absence of stage III cancer with residual disease <1 cm.

d In the DUO-O trial, stratification by surgical status included timing and outcome of cytoreductive surgery: absence of macroscopic residual disease after upfront surgery vs. all others. In the KEYLYNK-001 trial, presence or absence of residual tumor after primary debulking surgery or planned interval debulking.

e Patients were stratified by region of residence: North America, European Union, or rest of world.

f Patients were stratified based on whether their tumor PD-L1 combined positive score was <10 or ≥10.

g Categories included presence of residual disease vs. absence.

h Patients were stratified based on whether they had a history of primary debulking surgery or interval debulking surgery.

Stratification by surgical status is used in DUO-O (timing and outcome of cytoreductive surgery: absence or presence of macroscopic residual disease after upfront surgery) and KEYLYNK-001 (presence or absence of residual tumor after primary debulking surgery or planned interval debulking) but not FIRST because the latter excluded most patients with stage III R0 disease (with previously noted exceptions) after primary debulking surgery. The presence or absence of residual disease after frontline platinum-based therapy and the type of debulking surgery (primary or interval) are used to stratify randomization in ATHENA. These factors will need to be considered when interpreting data across trials, as they reflect biology of the disease and may affect outcomes. Another stratification factor of interest is concurrent use of bevacizumab (yes/no) at the investigator’s discretion in FIRST and KEYLYNK-001.

### Study treatment

Treatment regimens in each trial are described in Table 5 and Figure 1. In ATHENA, patients are randomly assigned for maintenance therapy (4:4:1:1) as follows: rucaparib plus intravenous nivolumab (arm A); rucaparib plus nivolumab-matched placebo (arm B); rucaparib-matched placebo plus nivolumab (arm C); or placebos matched to rucaparib and nivolumab (arm D). The trial consists of 2 independent comparisons assessing the effect of maintenance treatment with rucaparib monotherapy (ATHENA-MONO; rucaparib vs. placebo) in the frontline setting and the effect of combination therapy with nivolumab plus rucaparib (ATHENA–COMBO; rucaparib plus nivolumab vs. rucaparib plus nivolumab-matched placebo). Bevacizumab use is not permitted, although patients may have received it during first-line chemotherapy treatment.

**Table 5.** Treatment arms in phase 3 trials evaluating PARP inhibition plus immunotherapy for the first-line treatment of advanced epithelial ovarian cancer.

	First-line and first-line maintenance treatment settings			First-line maintenance treatment setting only
	FIRST (NCT03602859)	DUO-O (NCT03737643)	KEYLYNK-001 (NCT03740165)	ATHENA (NCT03522246)
**Maximum treatment duration**	156 weeks	104 weeks^a^	105 weeks^b^	104 weeks
**Comparator arm^c^**	Cycle 1: CP ± bev Q3W Cycles 2-6: CP ± bev Q3W + PBO IV Q3W Maintenance: niraparib QD + PBO IV Q6W ± bev Q3W	Patients with non-*BRCA*m tumors Cycles 1-6: CP + bev + PBO IV Q3W Cycle ≥7: bev + PBO IV Q3W + PBO PO BID	Cycle 1: CP ± bev Q3W Cycles 2-6: CP ± bev Q3W + PBO IV Q3W Cycle ≥7: PBO IV Q3W + PBO PO BID ± bev Q3W	Maintenance cycle 1: PBO PO BID Maintenance cycle ≥2: PBO PO BID + PBO IV Q4W
**Experimental arm 1**	Cycle 1: CP ± bev Q3W Cycles 2-6: CP ± bev Q3W + dostarlimab Q3W Maintenance: niraparib QD + dostarlimab Q6W ± bev Q3W	Patients with non-*BRCA*m tumors Cycles 1-6: CP + bev + durvalumab Q3W Cycle ≥7: bev + durvalumab Q3W + olaparib BID	Cycle 1: CP + bev Q3W Cycles 2-6: CP + pembrolizumab Q3W Cycle ≥7: pembrolizumab Q3W + olaparib BID	Maintenance cycle 1: rucaparib BID Maintenance cycle ≥2: rucaparib BID + nivolumab Q4W
**Experimental arm 2**	—	Patients with non-*BRCA*m tumors Cycles 1-6: CP + bev + durvalumab Q3W Cycle ≥7: bev + durvalumab Q3W + PBO PO BID	Cycle 1: CP ± bev Q3W Cycles 2-6: CP + pembrolizumab Q3W Cycle ≥7: pembrolizumab Q3W + PBO PO BID	Maintenance cycle 1: rucaparib BID Maintenance cycle ≥2: rucaparib BID + PBO IV Q4W
**Experimental arm 3**	—	Patients with *BRCA*m tumors^d^ Cycles 1-6: CP + durvalumab ± bev Q3W Cycle ≥7: durvalumab Q3W + olaparib BID ± bev Q3W	—	Maintenance cycle 1: PBO PO BID Maintenance cycle ≥2: PBO PO BID + nivolumab Q4W

Abbreviations: bev, bevacizumab; BID, twice daily; *BRCA*m, *BRCA* mutated; CP, carboplatin + paclitaxel; HRD, homologous recombination deficiency; IV, intravenous; PARP, poly(ADP–ribose) polymerase; PBO, placebo; PD-L1, programmed cell death-ligand 1; PO, orally; Q3W, every 3 weeks; Q4W, every 4 weeks; Q6W, every 6 weeks; QD, once daily.

a The maximum treatment duration for bev is 65 weeks.

b Patients receive pembrolizumab for a maximum of 35 3-week cycles and olaparib for a maximum of 29 3-week cycles.

c In the FIRST trial, the previous control treatment regimen was CP ± bev Q3W for cycle 1 followed by CP ± bev Q3W + PBO Q3W for cycles 2-6 and maintenance treatment with PBO QD + PBO Q6W ± bev Q3W. However, this arm was cancelled, and enrollment was discontinued after 193 patients had been admitted. These patients were removed from the blinding and offered maintenance niraparib. Data from this regimen will be limited.

d This patient cohort is open label and nonrandomized.

In KEYLYNK-001, patients are randomized (1:1:1) after 1 lead-in cycle of chemotherapy to receive (1) treatment with chemotherapy and pembrolizumab-matched placebo and maintenance with placebos for pembrolizumab and olaparib, (2) chemotherapy plus pembrolizumab and pembrolizumab plus olaparib-matched placebo for maintenance, or (3) chemotherapy plus pembrolizumab and pembrolizumab plus olaparib for maintenance. Use of bevacizumab is optional and determined per investigator’s discretion before randomization.

DUO-O includes 2 cohorts: patients with non-*BRCA*m tumors and those with *BRCA*m tumors. Patients with non-*BRCA*m tumors receive 1 cycle of chemotherapy ±bevacizumab before randomization (1:1:1) to 1 of 3 arms: treatment with chemotherapy plus bevacizumab and durvalumab-matched placebo and maintenance treatment with bevacizumab and placebos matched to durvalumab and olaparib (arm 1); chemotherapy with bevacizumab and durvalumab and bevacizumab plus durvalumab along with olaparib-matched placebo as maintenance (arm 2); or chemotherapy-bevacizumab-durvalumab combination and bevacizumab-durvalumab-olaparib combination for maintenance (arm 3). In the cohort of patients with *BRCA*m tumors, all patients receive open-label treatment with chemotherapy, bevacizumab, and durvalumab and maintenance treatment with a combination of bevacizumab, durvalumab, and olaparib. Bevacizumab use in this cohort is optional, according to the local practice.

In FIRST, all patients receive 1 cycle of chemotherapy ±bevacizumab before randomization (1:1) to either chemotherapy with dostarlimab-matched placebo or chemotherapy with dostarlimab. After completion, all patients receive maintenance therapy with niraparib (and continuation of either matched placebo or dostarlimab) ±bevacizumab. Initially, FIRST had an additional comparator arm with chemotherapy plus dostarlimab-matched placebo and placebos matched with niraparib and dostarlimab as maintenance. Following positive results from the PRIMA/ENGOT-OV26/GOG-3012^29^ and PAOLA-1/ENGOT-OV25^26^ trials, this arm was discontinued, in accordance with a protocol amendment,^64^ to ensure that all patients in the trial received the new standard of care (platinum-based chemotherapy ±bevacizumab plus maintenance treatment with a PARP inhibitor).

### Endpoints

Investigator-assessed PFS per Response Evaluation Criteria in Solid Tumors version 1.1 (RECIST v1.1; Table 6 and Figure 1) is the primary endpoint in all 4 trials, but the populations included in the primary analyses differ. In FIRST, PFS will be assessed in the intention-to-treat population. PFS in DUO-O is being evaluated hierarchically in patients with non–*BRCA*m/HRD-positive tumors followed by those with non-*BRCA*m tumors; the original endpoint in DUO-O was PFS in patients with non-*BRCA*m tumors. KEYLYNK-001 has two PFS endpoints in patients with PD-L1–positive tumors and in all patients; it is not clear from publicly available sources if these are coprimary endpoints or will be evaluated hierarchically. In ATHENA-COMBO, PFS is evaluated exclusively in the overall patient population. These between-trial differences will make outcome comparisons across trials challenging; however, the biomarker-driven analyses in some trials will be crucial in understanding the differential impact of these therapies on various subgroups. In FIRST, KEYLYNK-001, and ATHENA, PFS will also be assessed by blinded independent central review as a secondary endpoint. OS and safety are secondary endpoints in all trials; however, OS comparisons across trials will have to wait until after the data reach maturity. Health-related quality of life/patient-reported outcomes will be examined in all trials, as will efficacy endpoints related to subsequent treatment.

**Table 6.** Endpoints and biomarkers analyzed in phase 3 trials evaluating PARP inhibitor plus immunotherapy for the first-line treatment of advanced ovarian cancer.

	First-line and first-line maintenance treatment settings			First-line maintenance treatment setting only
	FIRST (NCT03602859)	DUO-O (NCT03737643)	KEYLYNK-001 (NCT03740165)	**ATHENA** ^65^ ** (NCT03522246)**
**Primary**	PFS in intention-to-treat population^a^	PFS in patients with non–t*BRCA*m HRD-positive tumors^c^ PFS in intention-to-treat population^60^	PFS in patients with PD-L1–positive tumors^e^ PFS in all patients	PFS in all patients
**Definition (time frame)**	Time from treatment randomization to the first documentation of progression or death (up to 6 years)^b^	Time from randomization to first progression or death (≈4 years)^d^	Time from randomization to first documented progressive disease or death (up to ≈57 months)	Time from randomization until disease progression (up to 7 years^f^)
**Measurement**	Investigator assessment per RECIST v1.1	Investigator assessment per modified RECIST v1.1	Investigator assessment per RECIST v1.1	Investigator assessment per RECIST v1.1
**Survival-­related secondary endpoints**	OS BICR-determined PFS per RECIST v1.1 criteria PFS2	Patients with non–*BRCA*m HRD-positive tumors: OS Patients with non-*BRCA*m tumors: PFS OS PFS2 Patients with *BRCA*m tumors: PFS PFS2	All patients: OS PFS per BICR Investigator-assessed PFS2 Patients with PDL-1–positive tumors: OS PFS per BICR Investigator-assessed PFS2	All patients: PFS per BICR OS
**OS definition (time frame)**	Time from randomization to death by any cause (up to 7 years)^b^	Time from randomization to death due to any cause (up to ≈7 years)	Time from randomization to death due to any cause (up to ≈6 years)	From enrollment to primary study completion (up to ≈10 years)
**Biomarkers**	HRR status evaluated using ctDNA PD-L1 expression ­(VENTANA [tumor area positivity >5%]) HRD	t*BRCA* and HRD status testing by Myriad MyChoice CDx (GIS ≥42)^60^	t*BRCA* status PD-L1 expression	t*BRCA* status LOH testing to determine HRD status (using FoundationOne CDx next-generation sequencing)

Abbreviations: BICR, blinded independent central review; *BRCA*m, *BRCA*-mutated; ctDNA, circulating tumor DNA; GIS, genomic instability status; HRD, homologous recombination deficiency; HRR, homologous recombination repair; LOH, loss of heterozygosity; OS, overall survival; PARP, poly(ADP–ribose) polymerase; PD-L1, programmed cell death-ligand 1; PFS, progression-free survival; PFS2, time to progression on subsequent therapy; RECIST v1.1, Response Evaluation Criteria in Solid Tumors version 1.1; t*BRCA*m, tumor *BRCA*-mutated.

a The primary endpoint was changed from PFS in patients with PD-L1–positive tumors and the intention-to-treat population.

b Original time frame was up to 5 years.

c Original primary endpoint was PFS in patients with non-t*BRCA*m tumors.

d Original time frame was approximately 6 years.

e Original primary endpoint was investigator-assessed PFS in all patients and OS in all patients.

f Originally up to approximately 10 years.

### Biomarker analysis

Based on publicly available information, a limited number of biomarkers are being evaluated across these studies (Table 6). FIRST is testing HRD status (*BRCA1/2* mutation and genomic instability) of patient tumor tissue samples using the MyChoice HRD plus CDx Assay (Myriad Genetics, Salt Lake City, UT, USA), PD-L1 status of patient tumor tissue samples using the VENTANA SP263 immunohistochemistry assay (Roche Diagnostics, Indianapolis, IN, USA), and circulating tumor DNA (ctDNA) for HRR mutations (*BRCA*m; *BRCA*wt HRR positive; *BRCA*wt HRR negative/not determined) using the ResBio ctDX-HRR assay (Resolution Bioscience, Inc, Kirkland, WA, USA). ATHENA uses the next-generation sequencing-based test (FoundationOne CDx; Foundation Medicine, Cambridge, MA, USA) to determine *BRCA* mutation status and the percentage of the genome with LOH; the trial also plans to assess other biomarkers, such as HRR genes other than *BRCA1/2*, PD-L1 expression, and tumor mutational burden.^65^ DUO-O is evaluating t*BRCA* and HRD status (MyChoice CDx test; Myriad Genetics, Salt Lake City, UT, USA),^60^^,^^66^ and KEYLYNK-001 plans to assess t*BRCA* status and PD-L1 expression. Currently, detailed information on assay type and methodology, as well as thresholds for biomarker evaluations (eg, PD-L1 status can be evaluated using tumor proportion score, tumor area positivity, or combined positive score thresholds), and where the positivity threshold is set (eg, combined positive score >5% vs. combined positive score >10%), are not available for all trials. The outcomes of biomarker testing may vary depending on the type of test used in the trial. The extent of these differences and how they affect interpretation of results from the trials remains to be seen but will be necessary to inform selection of patients who are most likely to benefit from these treatments.

## Discussion

PARP inhibitors are an established part of standard of care in ovarian cancer; however, some patients still experience suboptimal benefit from PARP inhibitor maintenance, particularly those with homologous recombination-proficient status.^67^ To date, PD-(L)1 inhibitor monotherapy has also not fulfilled the promise of additional benefit beyond current standard of care chemotherapy.^40^^,^^42^ The field is still awaiting results from the large phase 3 trials described above to assess benefit of a PARP inhibitor and an anti–PD(L)1 antibody combination therapy. Thus, results from FIRST, DUO-O, KEYLYNK-001, and ATHENA could reshape the ovarian cancer treatment landscape, if significant benefit is seen in any of the trials, specifically if there is an improvement in OS.

However, heterogeneity in patient populations and differences in stratification factors and treatment arms may complicate direct comparisons across trials. For example, ATHENA is the only trial that has a comparator arm in which patients receive no active drug during the maintenance phase; in the other 3 trials, patients in the comparator arm may receive at least 1 active drug in the maintenance phase. Thus, ATHENA is evaluating the addition of PD-1 inhibitor to the current standard of care in patients for whom bevacizumab is not indicated, but only for maintenance treatment. The primary aim of KEYLYNK-001 is to evaluate if the combination of pembrolizumab plus chemotherapy followed by continued pembrolizumab and maintenance olaparib is superior to chemotherapy alone. KEYLYNK-001 will be unable to assess if addition of a PD-(L)1 inhibitor to PARP inhibitor maintenance treatment improves outcomes in patients relative to the current standard of care. DUO-O is evaluating platinum-based chemotherapy plus bevacizumab (±durvalumab) followed by maintenance bevacizumab either as monotherapy, in combination with durvalumab, or in combination with durvalumab and olaparib. Similar to KEYLYNK-001, because the comparator arm of the DUO-O trial lacks a PARP inhibitor plus anti–PD-(L)1 placebo arm, DUO-O will be unable to assess if adding an anti–PD-(L)1 antibody to PARP inhibitor maintenance treatment improves outcomes in patients relative to the current standard of care. In contrast, comparison of the 2 treatment arms in FIRST will assess the effect of adding the PD-1 inhibitor dostarlimab to the current standard of care for advanced ovarian cancer.

The impact of bevacizumab in these trials will also be clinically interesting despite not being directly tested against a comparator arm in any trial; therefore, the individual contribution of bevacizumab to these combination regimens may be difficult to isolate. Clinician opinions on bevacizumab use in the current standard of care vary, and updated clinical trial data suggest that bevacizumab may only provide clinically relevant benefit in patients with high-risk disease^16–18^^,^^68^ or an unfavorable tumor intrinsic chemosensitivity.^69^^,^^70^ Data from these trials may better inform the role of bevacizumab in the evolving ovarian cancer treatment landscape.

Evaluation of endpoints in biomarker-defined subgroups, particularly those based on HRD and PD-(L)1 status, will be essential for guiding future treatment decisions and identifying patients most likely to benefit from combination therapy. However, intertrial variability may affect the interpretation and generalizability of the results. As an example, although addition of atezolizumab to platinum-based chemotherapy and bevacizumab in newly diagnosed stage III or IV ovarian cancer did not significantly improve PFS in patients with PD-L1–positive tumors (≥1% immune cells), exploratory analyses demonstrated an improvement in PFS when a threshold of PD-L1 immune cells ≥5% was used in the IMagyn050/GOG 3015/ENGOT-OV39 trial.^41^ Thus, patients with high PD-L1 expression may be more likely to benefit from this treatment regimen. Optimization of current biomarker use and identification of novel biomarkers are both essential to ensure that treatments are selective and can help improve patient outcomes. Therefore, there will be interest in identifying biomarkers associated with either negative or positive response in these trials, particularly on the predictive or prognostic role of PD-L1. Based on the publicly available information, prespecified analyses on predictive biomarkers may be limited; thus, predictive biomarker data will be largely limited to post hoc analyses.

## Conclusion

In this review, we show that although all 4 trials are evaluating the addition of immune checkpoint inhibitors to the first-line treatment of advanced ovarian cancer, in combination with platinum-based chemotherapy (±bevacizumab) and/or with PARP inhibitors for maintenance, there are differences in study design, patient population, and treatments that are likely to affect interpretation of the outcomes. Each trial has the potential to make major contributions to the treatment landscape for advanced ovarian cancer by providing data that will inform future updates to the standard of care. Data from ATHENA will show the effect of PARP inhibitor compared with placebo and the effect of PD-1 inhibitor plus a PARP inhibitor compared with PARP inhibitor alone as maintenance therapy, specifically in patients who had a response to the first-line chemotherapy. Results from KEYLYNK-001 will show the effect in high-risk non-*BRCA*m tumors, although the comparator arm is not the current standard of care because of the absence of PARP inhibitor maintenance. Similarly, results from DUO-O are also limited by the fact that the comparator arm does not include PARP inhibitor maintenance; therefore, it will not be possible to assess the benefit of adding PD-L1 inhibitor to the treatment regimen. In FIRST, after benefits of PARP inhibitor maintenance were demonstrated in phase 3 clinical trials, a protocol amendment was implemented to close the treatment arm where patients were not receiving a PARP inhibitor, thus ensuring the only comparator arm contained PARP inhibitor maintenance.^26^^,^^29^ Thus, data from FIRST will most likely inform the decision to add PD-(L)1 inhibitors to the current standard of care. Regardless of trial outcomes, the results of these phase 3 trials will help inform future trial designs and improve patient outcomes. Efforts should be taken to ensure that pretreatment and posttreatment biopsy samples or circulating tumor DNA samples are collected to accelerate translational research and make these translational data sets publicly available to facilitate collaboration, meta-analyses, and future studies.

## Supplementary Material

oyaf270_Supplementary_Data

## Data Availability

No new data were generated or analyzed in support of this research.
