# Supplementary material for: Phase 3 clinical trials evaluating poly(ADP-ribose) polymerase inhibition plus immunotherapy for first-line treatment of advanced ovarian cancer
Source: Oncologist. 2025 Sep 17;30(10):oyaf270. doi: 10.1093/oncolo/oyaf270 (PMC12578505; doi:10.1093/oncolo/oyaf270)
Supplement: oyaf270_Supplementary_Data [file oyaf270_supplementary_data.pdf]

Supplementary Material

This supplementary material has been provided by the authors to give readers additional information about their work.

Supplement to: Phase 3 Clinical Trials Evaluating Poly(ADP-Ribose) Polymerase Inhibition Plus Immunotherapy for First-Line Treatment of Advanced Ovarian Cancer

Supplementary Table 1. Key trial protocol updates after registration on ClinicalTrials.gov…………………..2

Supplementary Table 1. Key trial protocol updates after registration on ClinicalTrials.gov

**FIRST (NCT03602859)[a]**		
**Study record version**	**Version 1**	**Version 26**
**Enrollment, n**	**960[e]**	**1402[f]**
**Primary endpoint**	PFS in **patients with PD-L1-positive tumors** and **intention-to-treat population (≤5 y)**	PFS in **intention-to-treat population (≤7 y)**
**Survival-related secondary endpoints**	OS (**≤5 y**)	• OS (**≤6 y**)<br>• **BICR-determined PFS (≤6 y)**<br>• **PFS2 (≤6 y)**
**Key changes in inclusion criteria**	**Bolded sections were removed/updated**<br>• Nonmucinous epithelial ovarian (serous, endometrioid, clear cell, carcinosarcoma, and mixed pathologies) cancer<br>• Patients with stage III are eligible **if they meet the following criteria: high-risk stage IIIC disease and planning to receive neoadjuvant chemotherapy**<br>• Patients must provide a blood sample for research at screening<br>• Patient must provide an FFPE tumor tissue sample at screening for research	**Bolded sections were added/updated**<br>• **High-grade** nonmucinous epithelial ovarian (serous, endometrioid, clear cell, carcinosarcoma, and mixed pathologies), **fallopian tube, or peritoneal** cancer<br>• Patients with stage III are eligible if they **meet protocol defined criteria**<br>• Patients must provide a blood sample for **ctDNA HRR testing** at **prescreening or screening**<br>• Participant must provide a **minimum of 1 FFPE block slide at prescreening or screening for PD-L1, HRD testing**
**Key changes in exclusion criteria**	**Bolded sections were removed/updated**<br>• **Stage III patient with complete cytoreduction resection after primary debulking surgery (ie, no macroscopic residual disease, unless the patient has aggregate 5-cm extra-pelvic disease during primary debulking surgery**<br>• **Patient has a known condition, therapy, or laboratory abnormality that might confound the study results or interfere with the patient's participation for the full duration of the study treatment in the opinion of the Investigator**<br>• Patient is immunocompromised **Patients with splenectomy are allowed. Patients with well-controlled known HIV are allowed**	**Bolded sections were added/updated**<br>• **Patients has known active central nervous system metastases, carcinomatous meningitis, or both**<br>• **Patient has clinically significant cardiovascular disease**<br>• **Patient has a bowel obstruction by clinical symptoms or computed tomography scan, subocclusive mesenteric disease, abdominal or gastrointestinal fistula, gastrointestinal perforation, or intra-abdominal abscess**<br>• **Patient has any known history or current diagnosis of myelodysplastic syndrome or acute myeloid leukemia**<br>• **Prior treatment for high-grade nonmucinous epithelial ovarian, fallopian tube, or peritoneal cancer (immunotherapy, anticancer therapy, radiation therapy)**<br>• **Patient has an active autoimmune disease that has required systemic treatment in the past 2 y. Replacement therapy is not considered a form of**

		**systemic therapy (example, thyroid hormone or insulin)**<br>• **Patient has a diagnosis of immunodeficiency or is receiving systemic steroid therapy or any other form of systemic immunosuppressive therapy within 7 d before the first dose of study treatment.**
**Biomarkers**	None	HRR status evaluated using ctDNA<br>PD-L1 expression<br>HRD status
**Primary endpoint completion**	June 30, 2020	October 25, 2024
**Study completion**	July 31, 2023	June 22, 2026

**DUO-O (NCT03737643)[b]**		
**Study record version**	**Version 1**	**Version 56**
**Enrollment, n**	**1056[e]**	**1407[f]**
**Primary endpoint**	PFS in **intention-to-treat population (patients with non-t*BRCA*m tumors; ≈6 y)**	• PFS in **patients with non-t*BRCA*m HRD-positive tumors (≈4 y)**<br>• PFS in **intention-to-treat population** (**all patients with non-t*BRCA*m tumors; ≈4 y**)
**Survival-related secondary endpoints**	• OS and PFS2 in intention-to-treat population (patients with non-t*BRCA*m tumors; **≈6 y**)<br>• PFS and PFS2 in patients with t*BRCA*m tumors (**≈6 y**)	• **PFS in intention-to-treat population (patients with non-t*BRCA*m tumors; ≈4 y)**<br>• PFS2 in intention-to-treat (**patients with non-t*BRCA*m tumors; ≈7 y**) population<br>• **OS in patients with non-t*BRCA*m HRD-positive tumors and in all patients with non-t*BRCA* tumors (≈7 y)**<br>• PFS (**≈4 y**) and PFS2 in patients with t*BRCA*m tumors (**≈7 y**)
**Key changes in inclusion criteria**	**No changes in eligibility criteria**	
**Key changes in exclusion criteria**		
**Biomarkers**	**No changes in the biomarker analysis**	
**Primary endpoint completion**	May 18, 2022	March 28, 2025
**Study completion**	July 28, 2025	March 30, 2028

**KEYLYNK-001 (NCT03740165)[c]**		
**Study record version**	**Version 1**	**Version 126**
**Enrollment, n**	**1086[e]**	**1367[f]**
**Primary endpoint**	• PFS in all patients (**≈6 y**)<br>• **OS in all patients (≈6 y)**	• PFS in patients with **PD-L1–positive tumors (combined positive score ≥ 10)** (**≈57 months**)

		• PFS in all patients (≈57 months)
**Survival-related secondary endpoints**	• PFS per BICR in all patients **(≈6 y)**<br>• Investigator-assessed PFS2 in all patients (≈6.5 y)	• **OS in all patients and patients with PD-L1–positive tumors (combined positive score ≥ 10) (≈6 y)**<br>• PFS per BICR in all patients and in **patients with PD-L1–positive tumors (combined positive score ≥ 10) (≈4.75 y)**<br>• PFS2 (investigator-assessed) in all patients and **patients with PD-L1–positive tumors (combined positive score ≥ 10)** (≈6.5 y)
**Key changes in inclusion criteria**	**Bolded sections were removed/updated**<br>• Has histologically confirmed FIGO stage III or IV epithelial ovarian cancer (high-grade predominantly serous, endometrioid, carcinosarcoma, mixed mullerian with high-grade serous component, clear cell, or low-grade serous ovarian cancer), primary peritoneal cancer, or fallopian tube cancer<br>• Candidates for neoadjuvant chemotherapy, has a CA-125 (kU/L):CEA (ng/mL) ratio **>**25<br>• Has an ECOG PS of 0 or 1, as assessed within 7 d before initiating chemotherapy in the lead-in period and within **7** d before randomization	**Bolded sections were added/updated**<br>• Has histologically confirmed FIGO stage III or stage IV epithelial ovarian cancer (high-grade predominantly serous, endometrioid **(any grade),** carcinosarcoma, mixed mullerian with high-grade serous component, clear cell, or low-grade serous ovarian cancer), primary peritoneal cancer, or fallopian tube cancer<br>• Candidates for neoadjuvant chemotherapy, has a CA-125 (kU/L):CEA (ng/mL) ratio **≥**25<br>• Has an ECOG PS of 0 or 1, as assessed within 7 d before initiating chemotherapy in the lead-in period and within **3** d before **day 1 of cycle 1**
**Key changes in exclusion criteria**	**Bolded sections were removed/updated**<br>• Has known active central nervous system metastases and/or carcinomatous meningitis<br>• Has received CSFs (eg, G-CSF, GM-CSF, or recombinant erythropoietin) within **2 wk prerandomization**<br>• Has had surgery to treat borderline tumors, early-stage EOC, or fallopian tube cancer <6 mo prescreening	**Bolded sections were added/updated**<br>• Has known active central nervous system metastases and/or carcinomatous meningitis. **Patients with brain metastases may participate provided they were previously treated (except with chemotherapy) and are radiologically stable, clinically stable, and no steroids were used for the management of symptoms related to brain metastases within 14 d prerandomization. Stable brain metastases should be established before the first dose of study medication lead-in chemotherapy**<br>• Has received CSFs (eg, G-CSF, GM-CSF, or recombinant erythropoietin) within **4 wk before receiving chemotherapy during the lead-in period**<br>• Has had surgery to treat borderline tumors, early-stage epithelial ovarian cancer, or **early-stage** fallopian tube cancer <6 mo prescreening.

	• Has a known history of infection with HBV or known active infection with HCV.<br>• Has received prior treatment for **advanced or metastatic** ovarian cancer, including radiation or systemic anticancer therapy (eg, chemotherapy, hormonal therapy, immunotherapy, investigational therapy)<br>• Has intraperitoneal chemotherapy planned as first-line therapy<br>• Has received a live vaccine within 30 d before the first dose of study treatment<br>• Has severe hypersensitivity (grade ≥3) **to the study treatments** and/or any of their excipients<br>• Is currently receiving either strong (eg, phenobarbital, **enzalutamide**, phenytoin, rifampicin, rifabutin, rifapentine, carbamazepine, nevirapine, and St John's Wort) or moderate (eg, bosentan, efavirenz, modafinil) inducers of CYP3A4 that cannot be discontinued for the duration of the study<br>• Either has had major surgery within **2 wk** of randomization or has not recovered from any effects of any major surgery<br>• **Has received whole blood transfusions in the last 120 d prerandomization**	• Has a known history of HBV **(defined as HBsAg reactive)** or known active HCV **(defined as detectable HCV RNA [qualitative]) infection. Testing for HBV or HCV is required at screening only if mandated by local health authority. Note: Patients who have a history of HBV but are HBsAg negative are eligible for the study**<br>• Has received prior treatment for **any stage** of ovarian cancer, including radiation or systemic anticancer therapy (eg, chemotherapy, hormonal therapy, immunotherapy, investigational therapy)<br>• Has intraperitoneal chemotherapy planned **or has been administered** as first-line therapy<br>• Has received a live vaccine within 30 d before the first dose of study treatment on **day 1 of cycle 1**<br>• Has severe hypersensitivity (grade ≥3) **to pembrolizumab, olaparib, carboplatin, paclitaxel or bevacizumab (if using)** and/or any of their excipients<br>• Is currently receiving either strong (eg, phenobarbital, phenytoin, rifampicin, rifabutin, rifapentine, carbamazepine, nevirapine, and St John's Wort) or moderate (eg, bosentan, efavirenz, modafinil) inducers of CYP3A4 that cannot be discontinued for the duration of the study<br>• Either has had major surgery within **3 wk** of randomization or has not recovered from any effects of any major surgery<br>• **Has uncontrolled hypertension**
**Biomarkers**	**No changes in the biomarker analysis**	
**Primary endpoint completion**	August 8, 2025	August 26, 2024
**Study completion**	August 8, 2025	May 29, 2026

**ATHENA (NCT03522246)[d]**		
**Study record version**	**Version 1**	**Version 19**
**Enrollment, n**	1012[e]	1000[f]
**Primary endpoint**	Investigator-assessed PFS (up to approximately 10 y)	Investigator-assessed PFS (up to approximately 7 y)

**Survival-related secondary endpoints**	**No changes in survival-related secondary endpoint analyses**	
**Key changes in inclusion criteria**		**Bolded sections were added/updated**<br>• **Patients must be 20 years of age to consent in Japan, Taiwan, and South Korea; in all other participating countries, patients must be 18 years of age to consent**
**Key changes in exclusion criteria**	**No changes in the key exclusion criteria**	
**Biomarkers**	**No changes in the biomarker analysis**	
**Primary endpoint completion**	December 30, 2024	May 20, 2024
**Study completion**	December 30, 2030	December 30, 2030

Abbreviations: BICR, blinded independent central review; CA-125; cancer antigen 125; CEA, carcinoembryonic antigen; ctDNA, circulating tumor DNA; CYP3A4, cytochrome P450 family 3 subfamily A member 4; ECOG PS, Eastern Cooperative Oncology Group performance status; FFPE, formalin-fixed paraffin-embedded; FIGO, International Federation of Gynecology and Obstetrics; G-CSF, granulocyte colony-stimulating factor; GM-CSF, granulocyte-macrophage colony-stimulating factor; HBV, hepatitis B virus, HBsAg, hepatitis B surface antigen; HCV, hepatitis C virus; HRD, homologous recombination deficiency; HRR, homologous recombination repair; OS, overall survival; PARP, poly(adenosine-ribose) polymerase; PD-L1, programmed death-ligand 1; PFS, progression-free survival; PFS2, time to progression on subsequent therapy; t*BRCA*m, tumor *BRCA*-mutated.

[a]Comparisons made between version 1 (July 18, 2018) and version 26 (August 14, 2024).
[b]Comparisons made between version 1 (November 8, 2018) and version 56 (October 30, 2024).
[c]Comparisons made between version 1 (November 12, 2018) and version 126 (November 15, 2024).
[d]Comparisons made between version 1 (April 30, 2018) and version 19 (July 10, 2024).
[e]Original estimated enrollment in version 1 filed on ClinicalTrials.gov
[f]Actual enrollment in indicated version on ClinicalTrials.gov
